# Future Challenges in Cancer Resistance to Immunotherapy

**DOI:** 10.3390/cancers12040935

**Published:** 2020-04-10

**Authors:** Marit J. van Elsas, Thorbald van Hall, Sjoerd H. van der Burg

**Affiliations:** Department of Medical Oncology, Oncode Institute, Leiden University Medical Center, 2300RC Leiden, The Netherlands; m.j.van_elsas@lumc.nl (M.J.v.E.); T.van_hall@lumc.nl (T.v.H.)

**Keywords:** immunotherapy, primary resistance, secondary resistance

## Abstract

Cancer immunotherapies, including checkpoint inhibitors, adoptive T cell transfer and therapeutic cancer vaccines, have shown promising response rates in clinical trials. Unfortunately, there is an increasing number of patients in which initially regressing tumors start to regrow due to an immunotherapy-driven acquired resistance. Studies on the underlying mechanisms reveal that these can be similar to well-known tumor intrinsic and extrinsic primary resistance factors that precluded the majority of patients from responding to immunotherapy in the first place. Here, we discuss primary and secondary immune resistance and point at strategies to identify potential new mechanisms of immune evasion. Ultimately, this may lead to improved immunotherapy strategies with improved clinical outcomes.

## 1. Introduction

Despite major improvements in treatment, cancer remains a leading cause of death worldwide. With the global cancer burden being estimated at 18.1 million new cases and 9.6 million deaths in 2018, the need for improved treatment strategies is pressing [1]. In recent years, therapeutics that capitalize on the power of the host’s immune system to control and eliminate cancer have been developed. This causes a shift in the focus from the tumor itself, with therapeutic interventions being broad and aggressive (e.g., radiotherapy or chemotherapy), toward a more personalized and refined approach utilizing the immune system’s power and specificity. Several types of immunotherapeutic approaches have been developed, with checkpoint inhibition (CPI) and adoptive cell transfer (ACT) being the most successful, and therapeutic cancer vaccines starting to show the first signs of efficacy [2]. 

Immune checkpoints (ICs) function by modulating the immune response, in order to maintain self-tolerance and restrict the duration of the immune response [3]. T cell activation, through antigen recognition by the T cell receptor (TCR), is tightly regulated by the balance between co-stimulatory and co-inhibitory signals. Two of these co-inhibitory ICs, CTLA-4 and PD-1, have been studied extensively for their roles in cancer. CTLA-4 is a co-inhibitory molecule with the ability to directly inhibit T cell activation, as it counteracts CD28 co-stimulation by outcompeting its binding to their mutual ligands, CD80 and CD86 [4,5]. PD-1 is another inhibitory IC expressed on the cell surface of T cells, which is upregulated on T cells with an “exhausted” phenotype, following prolonged antigen exposure [6]. The blocking of co-inhibitory ICs to treat cancer has shown promising results in clinical trials [7,8,9]. Consequently, in 2011, the monoclonal antibody blocking CTLA-4 (Ipilimumab) was approved by the FDA for the treatment of advanced melanoma, followed by the approval of a PD-1-blocking monoclonal antibody (Pembrolizumab) for the treatment of melanoma in 2014, and the approval of a PD-L1 blocking antibody (Atezolizumab) for the treatment of urothelial carcinoma in 2016 [10,11,12]. Since then, several additional PD-1 and PD-L1 blocking antibodies have entered the market. Besides CTLA-4 and PD-1, there are several other IC targets currently under investigation, and the blocking of these inhibitory immune checkpoints, including NKG2A, is currently studied for its potential in creating novel therapeutic interventions [13,14,15]. Nevertheless, checkpoint inhibition is only effective in patients with a pre-existing tumor-reactive CD8+ T cell response, limiting its clinical applicability to certain tumor types and stages, if other means to provide tumor-reactive T cells are not applied [16].

One method to increase the number of tumor-reactive T cells is the adoptive transfer of ex vivo expanded tumor-infiltrating T cells (TILs) or transgenic T cells expressing a defined T cell receptor or chimeric antigen receptor (CAR T cells). The adoptive transfer of such cells has led to remarkable clinical responses, including the full regression of tumors [17,18,19]. Another therapeutic approach to increase the number of tumor-reactive T cells is the use of cancer vaccines. Therapeutic cancer vaccines aim to reinvigorate the patient’s T cell response to tumor-associated antigens (TAAs) or tumor-specific antigens (TSAs). Several vaccine platforms have been developed, including peptide, RNA and DNA vaccines, with encouraging efficacies as monotherapies in early disease stages or in combination with other immunotherapies in established tumors [20]. While TAAs have a broad applicability (multiple cancer types and stages), their origins as self-antigens may limit the efficacy of the responding T cells due to potential central tolerance mechanisms. This does not form a problem for the group of TSAs, comprising oncogenic virus-derived antigens and neoantigens, explaining why they should form very potent cancer vaccines. Indeed, several studies using genomic and bioinformatics approaches to design personalized neoantigen vaccines report a strong neoantigen-specific anti-tumor CD4+ and CD8+ T cell response correlated with tumor control in mice and humans [21,22,23,24,25]. Similarly, vaccines aiming to reinforce T cell reactivity to the highly oncogenic human papillomavirus type 16 (HPV16) encoded oncoproteins E6 and E7 not only induced strong HPV16-specific CD4+ and CD8+ T cell responses but also resulted in a high percentage of complete and partial regressions of HPV16-induced premalignant lesions [26,27,28,29]. It is important to note that the primary job of both the adoptive transfer of ex vivo expanded T cells and of therapeutic cancer vaccines is to amplify the tumor-reactive type 1 T cell pool and not to deal with immunosuppressive factors in the local tumor microenvironment (TME) known to be crucial [2]. Considering the fact that activated T cells start to express many co-inhibitory molecules, the combination of adoptively transferred T cells or vaccines with CPI holds a clear clinical advantage in keeping the tumor-reactive T cell response going [20]. As anticipated, clinical trials exploring the combination of cancer vaccines with CPI report improved clinical outcomes compared to those from monotherapies in multiple cancer types, suggesting a synergistic effect of these therapies [30,31,32]. Similar effects have been reported for adoptive cell transfer therapy with CPI [33,34]. 

## 2. Limitations of Immunotherapy

The shift in focus from the direct targeting of the cancer cell towards the stimulation of the anti-tumor response has resulted in encouraging clinical results in humans, but also comes with new problems. Despite promising overall response rates (ORR) for treatment with CPIs, tumor vaccines and ACT or a combination of these, response rates to immunotherapy vary greatly between tumor subtypes, depending partly on their immunogenicities [30,35,36,37,38,39,40,41,42,43,44]. Primary therapy resistance, defined as a lack of clinical benefit from immunotherapy on tumor growth, exists in a large proportion of patients. On top of this, and maybe even more importantly, a substantial percentage of tumors grow back after the initial response to therapy, even after deep regressions [30,45]. This process, known as secondary therapy resistance, most often occurs before tumors completely regress. For some tumor types, even after deep or complete regression, the risk of secondary resistance is very high [30,45]. Secondary resistance may appear as soon as 2 weeks after treatment initiation, despite the continuation of therapy. A series of examples of clinical trials where T cell-based immunotherapies result in primary and secondary resistance following different grades of initial response are listed in Table 1. This makes primary and secondary, or acquired, resistance one of the key factors responsible for curtailing overall survival rates in patients treated with immunotherapy. 

## 3. Extrinsic and Intrinsic Primary Resistance Mechanisms

### 3.1. Tumor Cell Extrinsic Primary Resistance Mechanisms

**Figure 1:**
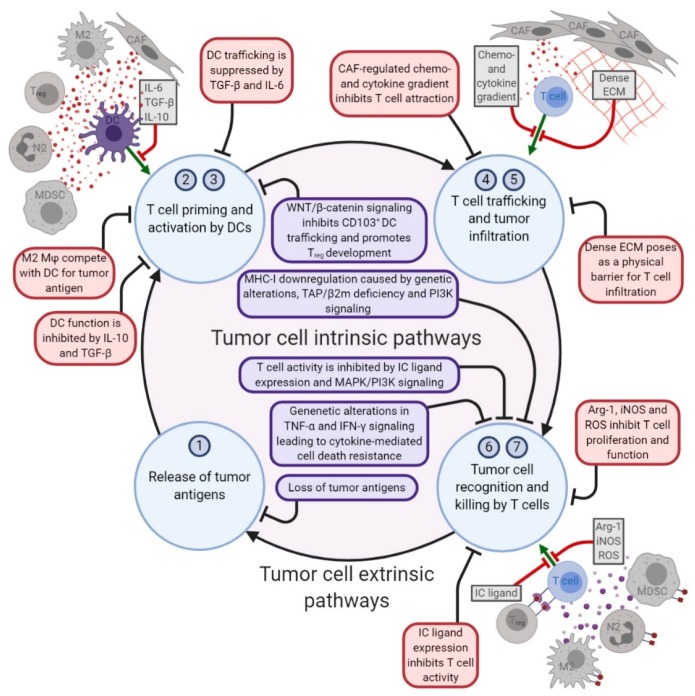
A simplified version of the cancer immunity cycle, adapted from Chen & Mellman [74] to show the tumor cell intrinsic and extrinsic pathways in T cell-based immunotherapy resistance. The numbers refer to steps in the original cancer immunity cycle. 1. The release of cancer cell antigens (cancer cell death); 2. Cancer antigen presentation (dendritic cells/APCs); 3. Priming and activation (Antigen Presenting Cells (APCs) & T cells); 4. The trafficking of T cells to tumors (Cytotoxic T Lymphocytes (CTLs)); 5. The infiltration of T cells into tumors (CTLs, endothelial cells); 6. The recognition of cancer cells by T cells (CTLs, cancer cells); 7. The killing of cancer cells (immune and cancer cells). *Created with BioRender.com*.

Factors driving therapy resistance can be either tumor cell intrinsic, determined by the traits of the tumor cell itself, or tumor cell extrinsic, involving the cells in the stroma of the TME (Figure 1). The migration of immunosuppressive cells to the TME can inhibit local immune cells from exerting their effector functions. Increased numbers of regulatory T (Treg) cells, myeloid derived suppressor cells (MDSCs), M2 macrophages and pro-tumor N2 neutrophils have all been linked to primary resistance against immunotherapies [46,47,48,49,50,51,52]. Although a complete overview of how these immunosuppressive cells exactly contribute to resistance against immunotherapy is still lacking, several underlying mechanisms have been described in detail (Figure 1). Firstly, the expression of ICs (including PD-L1 and CTLA-4) at the surface of these immune suppressive cells provides them with the means to inhibit local T cell activation directly [46,48,53,54]. Additionally, immunosuppressive mediators produced by these cells, including IL-10 and TGF-β, can enhance the establishment of a local network of immunosuppressive cells in the TME. For instance, TGF-β can polarize neutrophils to a pro-tumor, “N2-like” phenotype, thereby limiting the anti-cancer capacity of N1-like neutrophils [55]. Correspondingly, IL-10 and TGF-β can drive the differentiation of monocytes into M2-like tumor-associated macrophages (TAMs), which amongst their other suppressive actions, can also compete with local dendritic cells (DCs) for tumor antigens and consequently inhibit T cell priming [46,56,57,58]. In addition, IL-10 and TGF-β can limit local T cell priming through the suppression of both DC function and the proliferative capacity of T cells [59,60]. Alternatively, via the production of arginase-1 (Arg-1), inducible nitric oxide synthase (iNOS), reactive oxygen species (ROS), M2 macrophages, MDSCs and N2 neutrophils can inhibit T cell proliferation and function, while promoting the immunosuppressive properties of Treg cells [34,61,62,63,64,65]. Last but not least, TNF-α in the TME may also have a downside as it can bind to TNFR2, which is expressed by regulatory Treg cells and MDSCs to protect them from TNF-α induced death, while in the same way reducing the capacity of M1 macrophages to clear tumor cells [66]. Taken together, Treg cells, M2 macrophages, MDSCs and N2 neutrophils may suppress effector T cells systemically and in the TME, resulting in primary resistance mechanisms during cancer immunotherapy. 

In addition to tumor infiltrating immunosuppressive immune cells, the fibroblasts in tumors contribute to therapy resistance. One important driver of fibroblast activation in the TME is TGF-β, an immunosuppressive mediator found to interfere with the anti-tumor immune response. The TGF-β-driven activation of fibroblasts gives rise to a specific phenotype of immunomodulatory cancer-associated fibroblasts (CAFs). These CAFs, due to their abundance and heterogeneity, can orchestrate the response to cancer immunotherapy via several mechanisms (Figure 1). Firstly, through the release of TGF-β and IL-6, CAFs suppress the proliferation and trafficking capacity of antigen-presenting DCs, thereby interfering with tumor-directed T cell priming [67]. Secondly, through the tight regulation of the local chemokine- and cytokine-gradient, CAFs limit the attraction of T cells to the TME [68,69]. Moreover, TGF-β CAFs can remodel the composition of the extracellular matrix (ECM), resulting in a dense ECM network that poses a physical barrier to T cell infiltration [70]. Furthermore, CAFs can suppress the anti-tumor T cell response in the TME itself, through the upregulation of IC ligands on their cell surfaces [71]. Finally, tumor cells can “hijack” CAF metabolism to meet their metabolic needs, thereby shifting the balance in the metabolic competition between tumor cells and anti-tumor immune cells in favor of the tumor cells [72,73]. Together, these pathways drive CAF-dependent immune evasion and diminished responses to T cell targeted immunotherapies. 

### 3.2. Tumor Cell Intrinsic Primary Resistance Mechanisms

There are also several tumor intrinsic factors that mediate primary resistance against immunotherapy (Figure 1). The tumor intrinsic factors of primary resistance identified so far include 1) alterations in the antigen processing pathway; 2) a lack of tumor antigen expression; 3) the soft- and hard-wired loss of HLA expression; 4) alterations in the signaling pathways of MAPK, PI3K and WNT; 5) the constitutive expression of the ligands for IC (e.g., PD-L1 and HLA-E); and 6) resistance to TNF-α and IFN-γ mediated killing [75,76]. 

One way to identify these tumor cell intrinsic mechanisms are loss-of-function in vivo and in vitro screens. A study of primary resistance against a combination therapy of αPD-1 with a GM-CSF-secreting tumor cell vaccine applied a genetic in vivo CRISPR-Cas9 screen and identified several potential therapy-resistance genes [77]. Based on the top 50 most-depleted genes, four different signaling pathways associated with the sensitivity of tumor cells to treatment with immunotherapy were revealed, being TNF signaling/NFκB activation, the inhibition of kinase signaling, the ubiquitin-proteasome pathway and antigen processing and presentation [77]. For each of these pathways, a representative gene was selected, based on the highest cumulative score as ranked by the STARS algorithm. These genes were *Ripk1* for the TNF signaling/NFκB activation pathway, *Ptpn2* for the inhibition of kinase signaling pathway, *Stub1* for the ubiquitin-proteasome pathway and *H2-T23* for the antigen processing and presentation pathway [77]. Notably, *H2-T23* encodes Qa-1b (a mouse homolog of HLA-E), the ligand for the inhibitory receptor NKG2A, for which we demonstrated importance in mechanisms of acquired resistance to cancer vaccines, which may be alleviated by new antibodies to block NKG2A [14,15]. The involvement of the antigen processing and presentation pathway in immunotherapy resistance was confirmed in another CRISPR-based screen, which focused on the genes controlling HLA class I expression [78]. Here, IRF2 was identified as a rate limiting factor for TAP-mediated peptide transport to the endoplasmic reticulum and subsequent N-terminal trimming and thus antigen presentation [78]. IRF2 is frequently downregulated in tumors. TAP deficiency has been demonstrated in many cancer types and shown to correlate with disease progression and clinical outcomes [79,80,81]. Interestingly, tumor cells with such antigen processing defects still express MHC-I molecules, which then present T cell epitopes associated with impaired peptide processing (TEIPP) [82,83,84]. Priming TEIPP-specific T cells with vaccines to overcome acquired immune resistance has been proposed as a treatment strategy for tumors with impaired TAP expression, and this approach has been proven effective in inhibiting the outgrowth of immune-escaped tumors in mice [85,86]. Unexpectedly, the expression of MHC-II molecules was also detected on tumor cells and shown to correlate with T cell infiltration and the therapeutic response to CPI, indicating the presence of alternative antigen presentation pathways [87,88]. Notably, a lack of appropriate levels of tumor-specific antigen forms another important intrinsic resistance pathway against CPI [89]. In another screen, for key components determining the susceptibility of tumor cells to adoptively transferred effector cells, the GTPase Cdc42 was identified as a key factor in preventing CTL-induced cell death via MAPK signaling and posttranscriptional Bcl-2 stabilization [90]. Cdc42 is highly expressed in invasive cancers. Oncogenic MAPK signaling results in the production of immunosuppressive factors (e.g., VEGF, IL-6 and IL-10), which inhibit the proliferation and activation status of tumor-specific T cells and DCs [91]. In line with this, loss of the tumor suppressor gene *PTEN* has been shown to correlate with resistance against cancer immunotherapies, through the enhanced signaling of both the MAPK and PI3K signaling cascades [92,93]. Activation of the PI3K-AKT-mTOR pathway can contribute to therapy resistance by directly promoting tumor cell proliferation and survival, as well as the upregulation of PD-L1 cell surface expression, thereby inhibiting the function of local effector T cells [94]. Moreover, enhanced PI3K signaling via alternative AKT-independent pathways also acts on the antigen presenting pathway, as it results in the downregulation of HLA expression and escape from T cell recognition [95]. In addition to the MAPK and PI3K signaling cascades, the WNT/β-catenin pathway has also been implicated in resistance to cancer immunotherapies. A melanoma study on primary resistance against αPD-L1/αCTLA-4 antibody combination treatment revealed that the activation of the WNT/β-catenin pathway inhibits CD103+ DC-mediated T cell priming, resulting in a decreased infiltration of tumor-specific T cells to the TME [96]. Additionally, soluble melanoma-derived Wnt5a can alter local DC metabolism, leading to increased indoleamine 2,3-dioxygenase 1 (IDO) enzymatic activity and suppressed IL-6 and IL-12 production, thereby creating an immunosuppressive environment that promotes Treg development [97,98]. This IDO-driven Treg increase in the TME has been identified as a resistance mechanism against CTLA-4 and PD-1 CPI [98,99]. Notably, crosstalk between the MAPK, PI3K and WNT signaling pathways through the phosphorylation of cascade components occurs, making the targeting of these pathways to overcome immunotherapy resistance a complex ordeal [100,101]. 

Primary resistance to immunotherapy can also be the result of alterations in the TNF-α and IFN-γ signaling pathways protecting tumor cells against TNF-α- and IFN-γ-mediated cell growth regulation and death. A CRISPR-based in vitro and in vivo screen to identify mechanisms allowing tumor escape from CD8+ T cells and natural killer cells showed that the deletion of *Casp8*, *Tnfrsf1a* and *Ado* within the TNF-signaling pathway, or *Ifngr1/2*, *Jak1/2* and *Stat1* in the IFN-γ-signaling pathway, protected tumor cells against CD8+ T cell and/or NK cell-mediated killing and blunted the efficacy of anti-tumor responses in vivo [102]. In addition, the upregulation of the TNF receptor 2 (TNFR2) on tumor cells may foster tumor cell growth over TNFR1-induced killing after the binding of TNF-α [96]. Additionally, loss-of-function mutations or the downregulation of genes involved in the IFN-γ signaling pathway—such as *Ifngr1*, *Ifngr2*, *Jak1/2* and *Irf1*—were shown in patients who were irresponsive to αCTLA-4 antibody treatment and correlated to primary and adaptive resistance against αPD-L1 checkpoint blockade [103,104]. Primary resistance to CPI via alterations in antigen processing and presentation, as well as in responsiveness to IFN-γ signaling, was confirmed in a predictive biomarker study using single-cell RNA-sequencing (scRNA-seq) data from melanoma patients classified as untreated, CPI responsive or CPI resistant [105]. Taken together, the tumor cell extrinsic and intrinsic mechanisms driving immunotherapeutic resistance are versatile, yet tightly interwoven, making combination therapy an appealing therapeutic approach. It will be of interest to see if strategies that deal with these extrinsic and intrinsic primary resistance mechanisms will elucidate yet-unidentified mechanisms of resistance.

## 4. Secondary Resistance Mechanisms

It is important to realize that most factors determining initial resistance to immunotherapy are likely to also drive the occurrence of secondary immune escape. However, most of the studies have focused on the intrinsic resistance mechanisms.

Indeed, truncating mutations in JAK 1 and 2 were recently shown to form the basis for a lack of IFN-γ responsiveness in tumor cells and consequently for secondary resistance to CPI [104,106]. Interestingly, prolonged IFN-γ signaling is also one of the intrinsic mechanisms contributing to acquired resistance upon immunotherapy in humans [107]. This tight balance makes the interferon pathway a more challenging therapeutic target regarding acquired resistance against immunotherapy. Furthermore, a loss of antigen expression has been found in the form of epitope loss in CD19 after CAR T cell therapy and the loss of neoepitope expression after adoptive T cell therapy for melanoma [108,109]. One described mechanism driving the downregulation of (neo)antigen expression is promotor hypermethylation. However, this form of transcriptional alteration may only affect a small percentage of antigens, indicating that additional genomic and transcriptomic mechanisms are at play [110]. For example, low nutrient availability in the TME can lead to unresponsiveness to IFN-γ, resulting in decreased HLA class I expression [95]. In addition, an immunotherapy-driven loss of HLA class I expression due to decreased transcriptional expression of specific HLA class I genes was found after treatment with adoptively transferred T cells, anti-CTLA4 and anti-PD1, which can potentially be overcome by epigenetic modulators [111]. Moreover, the complete loss of HLA class I expression, due to the loss of the expression of the subunit beta-2 microglobulin (β2m), has also been found to be a secondary resistance mechanism in patients receiving αPD-1 CPI and after adoptive T cell transfer [112,113,114,115].

Due to limited research on the extrinsic factors fostering the development of secondary resistance to immunotherapy, there are only a few studies reporting the association between the attraction of immunosuppressive cells and the development of secondary resistance. In mice, the relapse of tumors after initial responses to combination therapy including dual CPI and radiotherapy was associated with an increase in Tregs in the TME [116]. These Tregs were phenotypically similar to those that were described to mediate primary resistance against immunotherapies, since RNAseq analysis of these cells revealed an increased expression of genes involved in TGF-β and IL-10 signaling. In another study, the increased expression of Tim-3 on the surface of Tregs in the TME was suggested to inhibit the local anti-tumor T cell response induced by mono CPI in combination with radiotherapy, leading to secondary resistance. Similarly, the accumulation of MDSCs in the TME was shown in patients that developed secondary resistance after initial responses to CPI [117]. These MDSCs were found to express PD-L1 and galectin-9, known ligands for the ICs PD-1 and Tim-3, respectively, providing them with the means to inhibit anti-tumor T cell function directly. 

In summary, the underlying mechanisms driving primary resistance against immunotherapy are abundant and diverse, and most factors determining the initial resistance to immunotherapy may also later drive the occurrence of secondary resistance.

## 5. Future Challenges and Conclusions

In view of the heterogeneity in background, tumor etiology and environmental conditions, it was to be expected that patients, even with the same type of cancer, would display highly variable responses to immunotherapy. The provided examples of therapy resistance indicate that the mechanisms underlying primary and secondary immune evasion can be versatile. Importantly, the complex system of immune regulation in the TME instinctively predicts that secondary resistance results from the interplay of multiple genetic factors, which may not always be identified in knockdown screens of single genes in tumor cells. In order to gain a complete understanding of the mechanisms at play, systematic analyses of therapy resistant tumors should be performed. 

**Figure 2:**
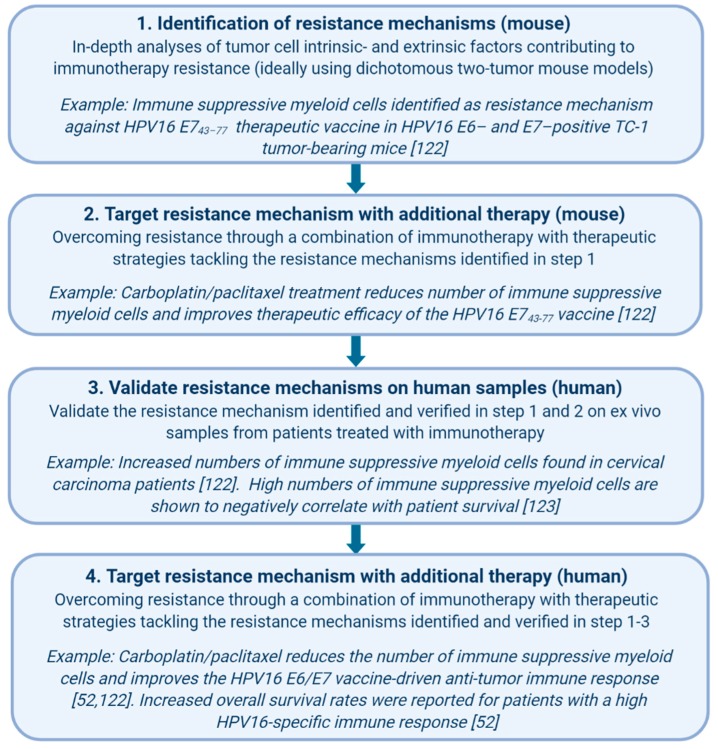
A workflow for the identification and validation of immunotherapy resistance mechanisms. *Created with BioRender.com*.

In order to delineate the underlying mechanisms of primary and secondary resistance, we advocate the investigation of so-called dichotomous responses in animal models. Even in the controlled conditions of inbred syngeneic mice and optimized treatment protocols, variation between animals is observed in terms of responsiveness to immunotherapy. This was described for the occurrence of secondary resistance in mice treated with the combination therapy α-CTLA-4 and αPD-1, after cDC1 anti-cancer vaccination and after combined treatment with CPI and an anti-tumor vaccine [118,119,120]. This dichotomous response makes mouse models an ideal alternative for new studies on the underlying mechanisms involved in secondary resistance to immunotherapy, and in some cases, they also may provide new leads to overcome this type of resistance. For instance, prolonged exposure to IFN-γ can result in acquired resistance to the combination of radiation therapy and α-CTLA-4, in line with human studies [107]. The application of genetically altered mice and tumor cell lines, as well as the application of other CPIs, revealed that this resistance was related to IFN-γ signaling pathway related events, including the upregulation of PD-L1, but also involved other regulatory pathways [107]. Well-defined extrinsic resistance mechanisms, as unraveled in mouse tumor models—with available research reagents, the depletion of antibodies and genetic knock-out systems—need to be confirmed operationally in cancer patients (Figure 2). This requires cancer samples from cohorts of refractory patients treated with the respective form of immunotherapy. Although challenging, we recently showed this to be feasible [121]. Immune suppressive myeloid cells were present at elevated levels in tumor-bearing mice and in patients treated with a therapeutic vaccine, resulting in a lower therapeutic efficacy and the suppression of spontaneous tumor-specific T cell reactivity, respectively [122]. Gemcitabine and the combination of carboplatin and paclitaxel both depleted MDSCs in mice, but only the latter was able to decrease the percentage of immune suppressive MDSCs in cancer patients with stronger spontaneous and vaccine-induced T cell reactivities, as well as result in clinical benefits [52,122,123]. Notably, the standard of care in humans is still directed at the tumor itself (chemotherapy, radiotherapy and surgery), and immunotherapies are, for now, mainly administered to patients with a history of at least one previous anti-cancer therapy. Along these same lines, we have to assume that each form of immunotherapy will yield its own unique resistance mechanism. This, together with human heterogeneity, should be taken into consideration when using mouse models to mirror the human cancer immunotherapy experience. Nevertheless, some acquired resistance mechanisms discovered in mouse tumor models are also operational in cancer patients, validating the use of mouse tumor models to identify not only secondary therapy mechanisms but also ways to overcome them (Figure 2) [106,107,111]. An important question, although harder to address, is what the factors within a tumor that determine the fate of the tumor during immunotherapy in the first place are. In general, this is easily overlooked since treatment responses in animal models are mostly studied at the stage in which regressor mice can be separated from non-regressors after therapy. At this late stage, the predictive factors that determine this outcome might already be lost. In a recent publication, this problem was acknowledged, and the authors proposed the use of a two-tumor model [124]. This allowed them to perform an in-depth ex vivo analysis of the dynamic tumor microenvironment of one surgically removed tumor, while the remaining tumor served for following therapeutic responses later on. While the authors focused on primary resistance against immunotherapy in this publication, the same approach could be used to identify the underlying mechanisms of acquired resistance. 

In conclusion, cancer immunotherapy has shown promising results in the clinic. Nevertheless, primary and secondary resistance occurs in the majority of patients, resulting in undesirable low rates of complete remission and overall survival. Studies that address these types of resistance and the underlying mechanisms are urgently needed in order to improve clinical outcomes, if targetable. This requires in-depth genetic studies of tumor intrinsic alterations mediating resistance, as well as of stromal cells in their TME, preferably in dichotomous two-tumor mouse models. Finally, the validation of primary and secondary resistance to immunotherapy in refractory patient cohorts will guide the development of optimal combinatorial therapies counteracting escape.

## Figures and Tables

**Table 1:** Examples of clinical trials resulting in acquired resistance during immunotherapy.

Therapy	Disease	Study Type	Patients Enrolled	RR			CCR	Relapse RR			Relapse CRR	Relapse Start
				10 <30%	30 <50%	50 <100%	100%	10 <30%	30 <50%	50 <100%	100%	
Pembrolizumab [35]	Advanced melanoma	Retrospective analysis	96	13	12 *	22 *	8 *	2/13	2/12	2/22	0/8	3 months
JS001 (PD-1 inhibitor) [36]	Advanced melanoma, urothelial cancer, renal cell cancer	Phase I clinical trial	36	5 *	3 *	4 *	1 *	2/5	1/3	0/4	-	8 weeks
Nivolumab OR Pembrolizumab [37]	Advanced NSCLC	Retrospective analysis	160	15	15	13	1	4/15	6/15	3/13	0/1	2 months
αPD-L1 antibody [38]	Melanoma NSCLC	Phase I clinical trial	41	7	5	5	1	2/7	3/5	2/5	0/1	6 weeks
Nivolumab [39]	Urothelial Cancer	Phase I/II clinical trial	74	8	5	12	3	5/8	3/5	2/12	0/3	6 weeks
Ipilimumab + Gemcitabine + Cisplatin [40]	Metastatic Urothelial cancer	Phase II clinical trial	36	1	4	9	8	1/1	3/4	3/9	6/8	6 weeks
Nivolumab + ISA 101 (SLP HPV16 vaccine) [30]	HPV16+ OPC, anal or cervical cancer	Phase II clinical trial	24	2	1 *	5 *	2 *	2/2	1/1	2/5	0/2	18 weeks
Pelareorep + Gemcitabine [41]	PDAC	Phase II clinical trial	29	7	1 *	0	0	3/7	0/1	-	-	1 month
siWT1 peptide vaccine + Gemcitabine [42]	PDAC	Phase II clinical trial	42	14	5	3	0	5/14	2/5	1/3	-	6 weeks
Adenoviral vector with IFNα2b gene + Celecoxib + chemotherapy [43]	MPM	Phase II clinical trial	40	7 *	10 *	8 *	0 *	2/7 *	3/10 *	1/8 *	-	6 weeks *
HPV+TILs + Cyclophosphamide + Fludarabine [44]	Cervical cancer, HPV^+^ cancer	Phase II clinical trial	29	6	16	3	2	2/6	9/16	0/3	0/2	1 month

RR 10 < 30% = a total tumor burden decline of 10–30% from baseline at some point during the study; RR 30 < 50% = a total tumor burden decline of 30–50% from baseline at some point during the study; RR 50 < 100% = a total tumor burden decline of 50–100% from baseline at some point during the study; CRR = a total tumor burden decline of 100% from baseline at some point during the study; Relapse = any total tumor burden decline followed by tumor outgrowth surpassing a size defined as RR (10–30%, 30–50%, 50–100% and 100%); Relapse start = the estimated time, from treatment initiation, at which tumors started to grow out again following the initial response. * Numbers verified by the authors; others were estimated based on published data when exact numbers were not provided.
